# Identification of critical factors that significantly affect the dose-response in mosquitoes irradiated as pupae

**DOI:** 10.1186/s13071-019-3698-y

**Published:** 2019-09-09

**Authors:** Hanano Yamada, Hamidou Maiga, Jose Juarez, Danilo De Oliveira Carvalho, Wadaka Mamai, Adel Ali, Nanwintoum Severin Bimbile-Somda, Andrew Gordon Parker, Dongjing Zhang, Jeremy Bouyer

**Affiliations:** 1 Insect Pest Control Laboratory, Joint FAO/IAEA Division of Nuclear Techniques in Food and Agriculture, International Atomic Energy Agency, Vienna International Centre, P.O. Box 100, 1400 Vienna, Austria; 2 Institute for Insectbiotechnology, Justus-Liebig-University Gießen, Winchester Str. 2, 35394 Gießen, Germany; 3 Key Laboratory of Tropical Disease Control of the Ministry of Education, Sun Yat-sen University - Michigan State University Joint Center of Vector Control for Tropical Diseases, Zhongshan School of Medicine, Sun Yat-Sen University, Guangzhou, 510080 Guangdong China

**Keywords:** *Aedes aegypti*, *Aedes albopictus*, *Anopheles arabiensis*, Hypoxia, Irradiation, Gammacell, Induced sterility, SIT

## Abstract

**Background:**

The sterile insect technique (SIT) for use against mosquitoes consists of several steps including the production of the target species in large numbers, the separation of males and females, the sterilization of the males, and the packing, transport and release of the sterile males at the target site. The sterility of the males is the basis of the technique; for this, efficient and standardized irradiation methods are needed to ensure that the required level of sterility is reliably and reproducibly achieved. While several reports have found that certain biological factors, handling methods and varying irradiation procedures can alter the level of induced sterility in insects, few studies exist in which the methodologies are adequately described and discussed for the reproductive sterilization of mosquitoes. Numerous irradiation studies on mosquito pupae have resulted in varying levels of sterility. Therefore, we initiated a series of small-scale experiments to first investigate variable parameters that may influence dose-response in mosquito pupae, and secondly, identify those factors that potentially have a significantly large effect and need further attention.

**Methods:**

In this study, we compiled the results of a series of experiments investigating variable parameters such as pupal age (*Aedes aegypti*), pupal size (*Ae. aegypti*), geographical origin of mosquito strains (*Ae. aegypti* and *Ae. albopictus*), exposure methods (in wet *versus* dry conditions, *Ae. albopictus*) and subsequently in low *versus* high oxygen environments [submerged in water (low O_2_ (< 5 %)] and in air [high O_2_ (~ 21 %)] on the radiosensitivity of male pupae (*Ae. aegypti*, *Ae. albopictus* and *Anopheles arabiensis*).

**Results:**

Results indicate that radiosensitvity of *Ae. aegypti* decreases with increasing pupal age (99% induced sterility in youngest pupae, compared to 93% in oldest pupae), but does not change with differences in pupal size (*P* = 0.94). Differing geographical origin of the same mosquito species did not result in variations in radiosensitivity in *Ae. aegypti* pupae [Brazil, Indonesia, France (La Reunion), Thailand] or *Ae. albopictus* [Italy, France (La Reunion)]. Differences in induced sterility were seen following irradiation of pupae that were in wet *versus* dry conditions, which led to further tests showing significant radioprotective effects of oxygen depletion during irradiation procedures in three tested mosquito species, as seen in other insects.

**Conclusions:**

These findings infer the necessity to further evaluate significant factors and reassess dose-response for mosquitoes with controlled variables to be able to formulate protocols to achieve reliable and reproducible levels of sterility for application in the frame of the SIT.
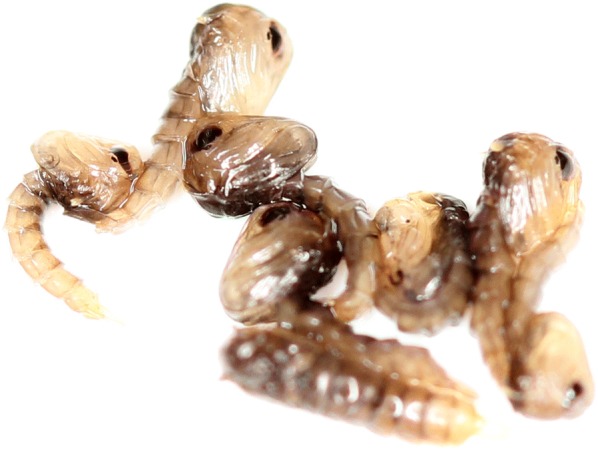

## Background

In the frame of the sterile insect technique (SIT) [1] and its application towards the management of medically important mosquito species, it is essential to standardize methods for the evaluation of radiation induced sterility in the males produced for releases. Consistent, reproducible and reliable irradiation methods are required to ensure that the target sterility level is reached for millions of male mosquitoes over time, so that no unknown levels of residual fertility can compromise the beneficial effects of the sterile males. It is also essential to balance the high sterility levels targeted, with optimal irradiation and handling protocols in efforts to improve male biological quality to minimize fitness costs and therefore maintain effectiveness in the field.

The first step for improvement for the irradiation procedures for mosquitoes is to harmonize materials and methods used and to find the causes for largely varying dose-response data currently found in the literature. Differences in radiosensitivity can be expected between families and even genera within the families of insects [2]; however, such variations in irradiation doses and resulting sterility within the same species beyond a “reasonable range” infers the effects of internal or external variables and these require evaluation and standardization before data on radiosterilizing doses are reliable for a given species.

To date, few publications exist reporting the effects of radiation on mosquito sterility compared to other insects of agricultural importance, and even fewer still adequately describe materials and methods used for the irradiation exposures, or biological variables that were controlled for (or not). No two experiments report the same method or the same result in terms of induced sterility (IS) at a given dose for a particular mosquito species. Important but scattered information, and incomplete descriptions of protocols used for the irradiation of mosquitoes have been described in some publications [3–15]; however, these have shown to be inadequate to reproduce the results consistently amongst research groups around the globe. Balestrino et al. [4] give a useful and detailed summary of past irradiation experiments for a variety of mosquito species, where the authors highlight that the results regarding induced sterility varies significantly amongst these reports. Many miss important factors such as verification of actual doses received by samples due to lack of dosimetry; few describe details of the sample preparation and handling methods, or are lacking information regarding other variable factors such as exact age, irradiation canisters and sample holding containers and handling methods, that may have affected the dose-response. However, there are several parameters that were shown to be important in these studies, and in studies involving other insect species, and these deserve a closer look on their own to assess their impact on dose-response in mosquitoes.

In radiation studies the primary parameter needing reliability and replicability is dose. For research (and operational) purposes, it is desirable to achieve doses as close as possible to the target dose with the smallest possible dose variation within the sample being irradiated. Dose rate varies within the available irradiation volume of any irradiator with the distance from the radiation source(s) and the attenuation of the radiation by absorption both in the sample material itself and in the chamber and sample holder material. The dose rate must, therefore, be measured throughout the sample being irradiated (or a suitable dummy material) for each load configuration (load size, shape and position within the radiation field) used to determine if the desired maximum dose variation will be exceeded. The load configuration can then be adjusted to bring the dose variation within the desired limits. Therefore, for all studies, the configuration must be standardized to assure that the dose reported, is indeed the true dose received by the sample.

Age has often been shown to have an effect on radiosensitivity. In general, the older the life stage, or the age within the life stages, the more radioresistant the insects become. This is because dividing cells are more susceptible to damage and developing stages of biological tissues are thus generally more sensitive than developed tissue [16]. Older pupae of several species of Tephritid fruit flies [17], Lepidoptera [18, 19] and *Glossina* spp. [20] have also been reported to be more radioresistant as pupal ages increase. Studies in different mosquito species give mixed reports, with different levels of significance given to pupal age as a factor affecting radiosensitivity [4, 10, 13].

In general, the sexes of arthropods have differential responses to irradiation. More often, females are more sensitive to irradiation than males [17, 21, 22], although there are some exceptions (such as in Glossinidae, males are more radiosensitive than females [23]). The enhanced radiosensitvity in females compared to males is also seen in some mosquito species, such as *An. arabiensis* [24], *Culex pipiens* [14], *Ae. aegypti* [52] and *Ae. albopictus* [4], where females cease to lay eggs altogether at doses of around 60–70, 70, 45 and 30 Gy, respectively.

Genetic differences representing geographical diversity could contribute to slight variations of radiosensitivity within the same species of insect but may not necessarily be the case. Only a few reports exist where such a difference has been described, and explanations for these inherent differences were hypothesized to have developed in response to external factors (such as altitude where the insects were reared) [25, 26]. Indeed, the important variable may have been the differences in size of the insects, resulting from differences in rearing. For mosquito irradiation, it is difficult to tell whether there may be differences in inherent radiosensitivity, as strains irradiated in different institutes, countries and using different protocols cannot be compared effectively. It is also important to keep in mind that different strains for the same species may have a slightly different level of natural sterility. Therefore, it is not useful to compare hatch rates, but rather the corrected hatch rates in reference to the control fertility, in other words, the induced sterility for all experiments assessing the effects on fertility.

Atmospheric conditions during irradiation, particularly differences in oxygen levels, have been shown to have significant impacts on dose-response in insects. Radiation effects are generally reduced in oxygen-poor environments (hypoxia) compared to oxygen-rich environments (normoxia), as radiation induces a chain of oxidative reactions. In the absence of oxygen, the free radicals may combine with hydrogen radicals, reducing the overall impact [27, 28]. The effect of hypoxia on dose-response has been well documented in other insects, particularly in agricultural pests such as the Mediterranean fruit fly (*Ceratitis capitata*), apple maggot (*Rhagoletis pomonella*), oriental fruit moth (*Grapholita molesta*), European corn borer (*Ostrinia nubilalis*) and plum curculio (*Conotrachelus nenuphar*), which were rendered more radioresistant in the absence of oxygen [29–31]. Some reports exist from the 1970s and 80s where atmospheric conditions affected dose-response in mosquitoes. Irradiation of *An. gambiae* (pupal stage), *Culex quinquefaciatus* (pupal and adult stages) and *Ae. aegypti*, all in nitrogen, showed that higher doses were needed to achieve the target sterility [6, 30, 32].

Seeing in other insects that there are numerous variables that have an impact on dose-response, we initiated a series of preliminary studies to investigate these factors with the aim to identify those that may have an important role in the irradiation of mosquito pupae, and to select the factors that may have significant effects in dose-response for further evaluation at a greater scale. This work compiles the results of these preliminary tests which were performed over a period of two years. The experimental methods change between experiments as new information regarding certain parameters became available and understanding of radiation-induced effects increased. It is for this reason that the sample sizes and number of repetitions, or selected doses vary in the different experiments. The long-term aim of this collected work is to acquire sufficient information to develop guidelines for the effective and reliable irradiation of mosquito pupae.

## Methods

### Mosquito strains

The *Ae. aegypti* strain originated from field collections in Juazeiro (Bahia), Brazil and were transferred to the Insect Pest Control Laboratory (IPCL) of the FAO/IAEA Agriculture and Biotechnology Laboratories, Seibersdorf, Austria from the insectary of Biofabrica Moscamed, Juazeiro, Brazil in 2016.

The *Ae. albopictus* strain originated from field collections in northern Italy and has been maintained under laboratory conditions at the Centro Agricoltura Ambiente, Bologna, Italy. The strain was transferred to the IPCL in 2012. Both the *Ae. albopictus* and *Ae. aegypti* strains have been maintained following the FAO/IAEA guidelines for the routine colony maintenance of *Aedes* mosquitoes [33].

The Dongola strain of *An. arabiensis*, originating from Dongola, Northern State, Sudan, was donated by the Tropical Medical Research Institute, Khartoum, Sudan in 2004 and has been maintained at the IPCL following the FAO/IAEA guidelines for the mass-rearing of *Anopheles* mosquitoes [34].

Four *Ae. aegypti* strains donated from La Reunion (France), Brazil, Thailand and Indonesia and two strains of *Ae. albopictus* from La Reunion (France) and Italy (Rimini), were used in the experiment assessing the effects of differential strain origin on radiosensitivity.

### The irradiator

**Fig. 1:**
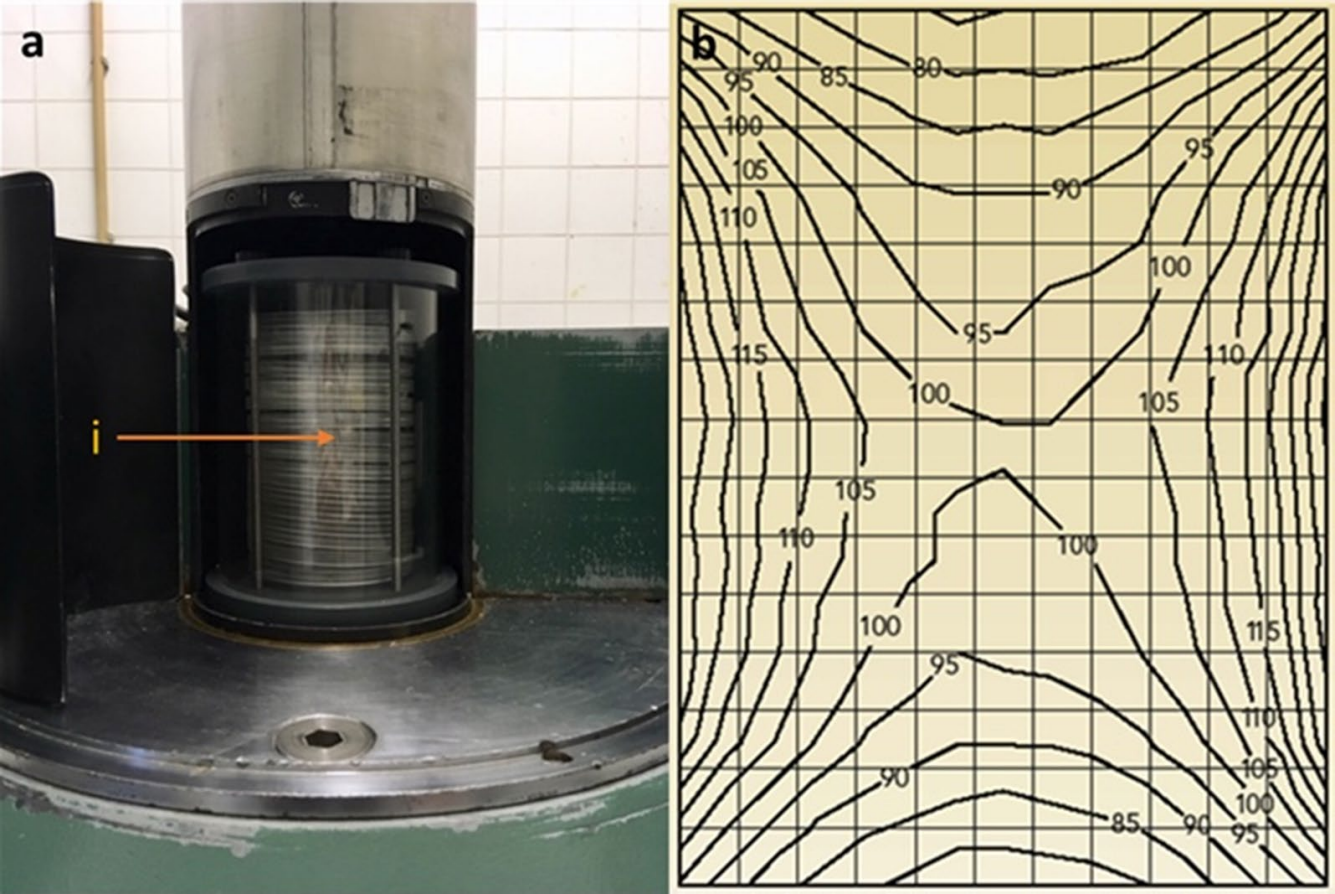
**a** A Gammacell 220 irradiation chamber containing a mosquito pupae holding canister consisting of stacked Petri dishes (pupae and dosimeters are in a central position). **b** Dose distribution map: a vertical section dose map of a GC220, with doses varying from 75 to 135% of the center dose (DUR = 1.8)

The irradiation device used in these experiments was a Gammacell 220 (Nordion Ltd, Kanata, Ontario, Canada). Over the course of the following experiments, the dose-rate of the source (Co60) decreased from 1.478 to 1.296 Gy/s at the time of the last experiment. The dose rate within the chamber volume varies substantially, with the lowest dose at the top and bottom of the chamber and the highest at the middle periphery (Fig. 1). The overall dose uniformity ratio (DUR) is 1.8, but the dose rate varies least in the middle of the chamber. The custom-made irradiation canister for mosquito pupae irradiation improves the DUR to approximately 1.3 in its interior (Fig. 1). To ensure that pupae samples did not receive significantly varying doses between experiments, the samples were always placed in the same center position on the centremost Petri dish, to further improve the DUR to below 1.1 to ensure consistency.

### Dosimetry

To enable comparisons of the doses applied in different experiments and facilities, a suitable dosimetry system calibrated with traceability to a national standard is required [35]. The calibration provides both a value for the dose received and an associated uncertainty, so that the confidence interval of the measurement can be calculated. A dosimetry system was used in all experiments to verify the dose received by the batches based on Gafchromic HD-V2 and MD-V3 film (Ashland Advanced Materials, Bridgewater NJ, USA) [35]. Three films of either HD film (for doses *>* 60 Gy) or MD film (for *<* 60 Gy) were packed in aluminium envelopes (to avoid getting wet) and placed directly above and below the pupae samples. The temperature near the sample and films was measured before and after radiation exposure. Films were read with an optical density reader after 24 h of development.

### Effects of strain geographical origin

One variable which is present in all historic and current irradiation studies around the globe is the geographical origin of the mosquito strains. To assess whether this factor could be the reason why different studies by different researchers result in inconsistent results for induced sterility, we compared the dose-response in *Ae. aegypti* and *Ae. albopictus* strains that were donated from different geographical regions. *Aedes aegypti* strains from Brazil, Indonesia, France (La Reunion) and Thailand, and *Ae. albopictus* strains from Italy and France (La Reunion) were assessed.

Approximately 2000 eggs were used for each strain. Larvae were reared according to the “Guidelines for Routine Colony Maintenance of *Aedes* mosquitoes” [33]. Pupae were collected on the sixth day at either 09:00 h, 12:00 h or 15:00 h to obtain > 40-h-old pupae by the time of irradiation. Male and female pupae were separated based on size and confirmed under a microscope. For each strain, female pupae were placed in individual tubes for emergence to ensure virginity and were kept for mating. Male pupae were split into two treatments (control and irradiation) with 50 pupae per treatment, per strain, and with three replicates each. The treatment groups of the 4 strains of *Ae. aegypti*, (and 2 strains of *Ae. albopictus*) were irradiated at 40 Gy in a custom-made Petri dish containing 4 wells equidistant from the center point, which was placed in the middle of the irradiation canister, with excess water removed, and in the center of the Gammacell chamber (Fig. 1a). For each repetition, the strains were rotated among the wells in the Petri dish.

A total of 40 male pupae were kept for emergence in cages and were mated to virgin females at a 1:1 ratio. Females were offered 3 blood meals (defibrinated porcine blood) to ensure a high overall blood-feeding rate. Females were then separated and placed into individual tubes with distilled water and oviposition paper for egging. Females were allowed to lay eggs over 4 days.

The eggs were then collected, dried and hatched using hatching solution [0.7 l of deionized water, 0.25 g of CM 0001 Nutrient Broth (Oxoid, Hampshire, UK) and 0.05 g of yeast] for 24 h as described by Zheng et al [36]. The number of viable L1 larvae and egg hatch rate was determined under a stereomicroscope and induced sterility calculated.

### Effects of pupal age on radiosensitivity in *Ae. aegypti*

The effect of age in *Ae. aegypti* pupae has been assessed and reported for 2 broad age groups (young, 19–23 h; and old, 42–46 h) [37]. To get a better picture of the relationship between pupal age and induced sterility, 5 random age groups (45–50 h; 42–45 h; 26–42 h; 20–24 h; and 10–24 h) were irradiated in Petri dishes, with excess water removed (but pupae still damp), at a fixed dose (40 Gy) and induced sterility was assessed and compared. A diagnostic dose of 40 Gy was selected, aiming to achieve around 90% induced sterility to be able to see increased or decreased effects without arriving at 0% hatching. Age groups were selected according to when 40–50 male pupae (2 times 20 per technical repetition) became available and could be collected. The 2 repetitions involved pupae from the same cohort; however, each rep was irradiated in separate irradiation events and were mated in separate cages, and resulting egg batches were analysed separately. Female pupae were sexed by size using a glass pupal sorter, and then were tubed individually to ensure sex and virginity. Virgin females were added to each cage containing 20 males at a 1:1 ratio and were allowed to mate for 3 days before being offered blood meals on 2 consecutive days to ensure a high overall blood-feeding rate. Oviposition cups lined with germination paper and filled half way up with water were added to the cages 3 days after the first blood meal. Eggs were collected *en masse* and were matured (slow-dried over 4 days) and stored for 10 days before hatching. Hatched larvae were counted, and the remaining unhatched eggs were bleached for *c.* 10 min in a 6% sodium hypochlorite solution to identify any remaining unhatched, fertile eggs. In addition, adult male longevity was followed in both repetitions by removing all dead males every 2–3 days until all males were deceased.

### Effects of pupal size on radiosensitivity in *Ae. aegypti*

*Aedes aegypti* larvae from 2 different cohorts were split and reared in rearing trays at either a very high density (6 larvae/ml) or very low density (1 larva/ml) to obtain small (*<* 0.900 mm) and large pupae (*>* 1.100 mm), respectively. Amounts of diet per larva were equal for all trays in both groups. The pupae were collected over a 6-h period to ensure that all pupae were between 24 and 30 h-old at the time of irradiation. The pupae were counted into 4 groups of 30 for the 4 repetitions per size. All groups were irradiated in Petri dishes with excess water removed, at a diagnostic dose of 30 Gy. This dose was chosen as the results for the previous experiment using 40 Gy came close to 99% induced sterility for some treatment groups and full sterility needed to be avoided as a 0% hatching is not comparable. Irradiated males and controls were mated to virgin females (prepared as described in the previous section “Effects of pupal age on radiosensitivity”) and the resultant eggs were collected *en masse* and were checked for fertility (also as previously described in the same section).

### Effects of sample preparation and ambient conditions during exposure

*Aedes albopictus* larvae were reared as described in the “strain geographical origin” section. Pupae aged 24–30 h were then divided and prepared to be irradiated in either “dry conditions” or “wet conditions” at 40 Gy. For the “dry” treatment, pupae were dried by placing them onto a paper towel to remove all water. For the “wet” treatment, pupae were placed in a 15 ml falcon tube such that they were swimming/floating in 1 ml of water. The tube containing pupae was held in place in the middle of the irradiation canister (Fig. 1) for irradiation, so that the location of the pupae was the same for both treatments. For each treatment, batches of 100 male pupae were irradiated. Three repetitions were performed for each treatment. Controls consisted of the same number of pupae that were not irradiated. Mating, blood-feeding, egging and egg hatch check were performed as described above in the “strain geographical origin” experiment.

### Effects of atmospheric conditions on induced sterility in *Ae. aegypti*, *Ae. albopictus* and *An. arabiensis*

Following the previous experiment assessing handling and sample preparation methods, the ambient conditions were further controlled by inducing a low oxygen environment [using water, which is known to have an O_2_ level of 8% and lower at temperatures over 25 °C, (https://www.engineeringtoolbox.com/air-solubility-water-d_639.html)] during irradiation and compared to an environment of air (which is known to have levels of oxygen at around 21%, defined as a normoxic condition).

#### Sample preparation

Pupae of all 3 species were collected in 4-h windows to ensure uniform pupal age of 40–44 h for both *Aedes* strains, and 20–24 h for *An. arabiensis*. *Aedes* pupae were sexed based on pupal size dimorphism using a glass pupal sorter [38] and sex was verified under a stereomicroscope. Pupae of *An. arabiensis* were sexed visually using a stereomicroscope. Males were kept for treatment and females were placed in individual tubes for emergence to ensure sex and virginity for later mating. To establish a low oxygen environment, male pupae were counted into batches of 160 and were placed in 1.5 ml Eppendorf tubes containing water. This pupal density was selected according to observations by D. Zhang in which pupae at this density, submerged in water showed a decreased radio-sensitivity compared to other reports on irradiation of the same strain. For the normoxic conditions, pupae were placed in the center (2 cm diameter ring made with a hot-melt adhesive) of 100 × 15 mm standard Petri dishes for irradiation. Excess water was removed so that the pupae were surrounded by air. The Eppendorf tubes were closed 30 min prior to irradiation treatment to establish hypoxia (low oxygen environment) by allowing depletion of oxygen by respiration through the cuticles of the contained pupae, as was seen by Zhang (unpublished data). Both the pupae in hypoxic and normoxic treatment groups were irradiated at the same time to ensure equal dose. Four biological repetitions were performed for both *Ae. aegypti* and *Ae. albopictus*, and 3 repetitions for *An. arabiensis*, each with 3 technical repetitions.

#### Irradiation

Radiation treatments and controls were performed in either hypoxic or normoxic conditions. A diagnostic dose was selected according to the expected dose required to induce less than 100% sterility in each strain and each treatment: 70, 35 and 95 Gy for *Ae. aegypti*, *Ae. albopictus* and *An. arabiensis*, respectively.

#### Assessment of induced sterility

Following irradiation, 50 males were randomly selected from each treatment group and were placed in a 15 × 15 × 15 cm BugDorm cage (MegaView Science Co. Ltd., Taichung, Taiwan) for emergence. Fifty virgin females prepared as described above (and of the same age) were added to each cage and were allowed to mate for 3 days. All batches were provided with 2 blood meals on consecutive days to increase overall blood-feeding rates following the mating period. Oviposition cups containing water and egg papers (germination paper) were added to each cage for egg collection *en masse* following routine rearing protocols [33]. Egg papers from *Ae. aegypti* and *Ae. albopictus* were collected, matured (slow-dried over 4 days) and stored for 10 days before hatching. Hatch rates were determined under the stereomicroscope. Unhatched eggs were bleached for *c.* 10 min in a 6% sodium hypochlorite solution to identify any remaining unhatched, but fertile eggs. The *An. arabiensis* eggs were collected and hatched the same day. The total number of eggs and the number of L1 larvae were counted for each treatment group to derive the hatch rate. The obtained hatch rate was further verified by counting the number of hatched and un-hatched eggs using a stereomicroscope and the induced sterility was calculated.

### Statistics

The residual fertility (RF) was calculated as a percentage of the control fertility of each treatment group (RF = HR_tx_/HR_c_ × 100), where HR_tx_ is the hatch rate of the treatment (tx) group, and HR_c_ is the hatch rate of the control (c) group. Induced sterility (IS) was calculated by subtracting the RF from 100%.

Statistical analyses were performed using Microsoft Excel (v.16.0, Microsoft, Redmond, WA, USA), GraphPad Prism v.5.0 (Graphpad Software, La Jolla, CA, USA; http://www.graphpad.com) and R v.3.5.2 [39].

The effect of geographical origin on induced sterility was analyzed using a Gaussian linear mixed-effects model fit by maximum likelihood, with strain/country as fixed effect and repetitions as a random effect.

A Pearson’s correlation coefficient was used to detect the linear correlation of pupal age and the induced sterility. ANOVA was performed to test the effect of pupal age on induced sterility followed by Tukey’s multiple comparisons of means to compare each pair of age group. The longevity of males from all age groups was analyzed using Kaplan–Meier survival analyses. The log-rank (Mantel–Cox) test was used to compare the level of survival between different age groups. To account for the multiplicity of comparisons the Bonferroni correction method was applied for each pair of age group. For this test, alpha levels were corrected to *P* < 0.0033.

A Gaussian linear mixed-effects model fit by maximum likelihood, with pupal size as fixed effect and repetitions as a random effect was used to analyze the effect of pupal size on induced sterility. The same analysis was used to test the effects of sample preparation and ambient conditions during exposure on induced sterility.

A binomial linear mixed effect models were used to analyze the impact of hypoxia on the hatching rate (R). The treatment regimens for irradiation were then used as fixed effects and the repetitions as random effects. The significance of fixed effects was tested using the likelihood ratio test [40, 41]

The best model in all analyses was selected based on the lowest corrected Akaike information criterion (AICc), and the significance of fixed effects was tested using the likelihood ratio test. All significant differences are based on *P* < 0.05.

## Results

### Dosimetry

The dosimetry confirmed that all doses received lay within the 5% confidence interval of the calibration.

### Strain geographical origin

**Fig. 2:**
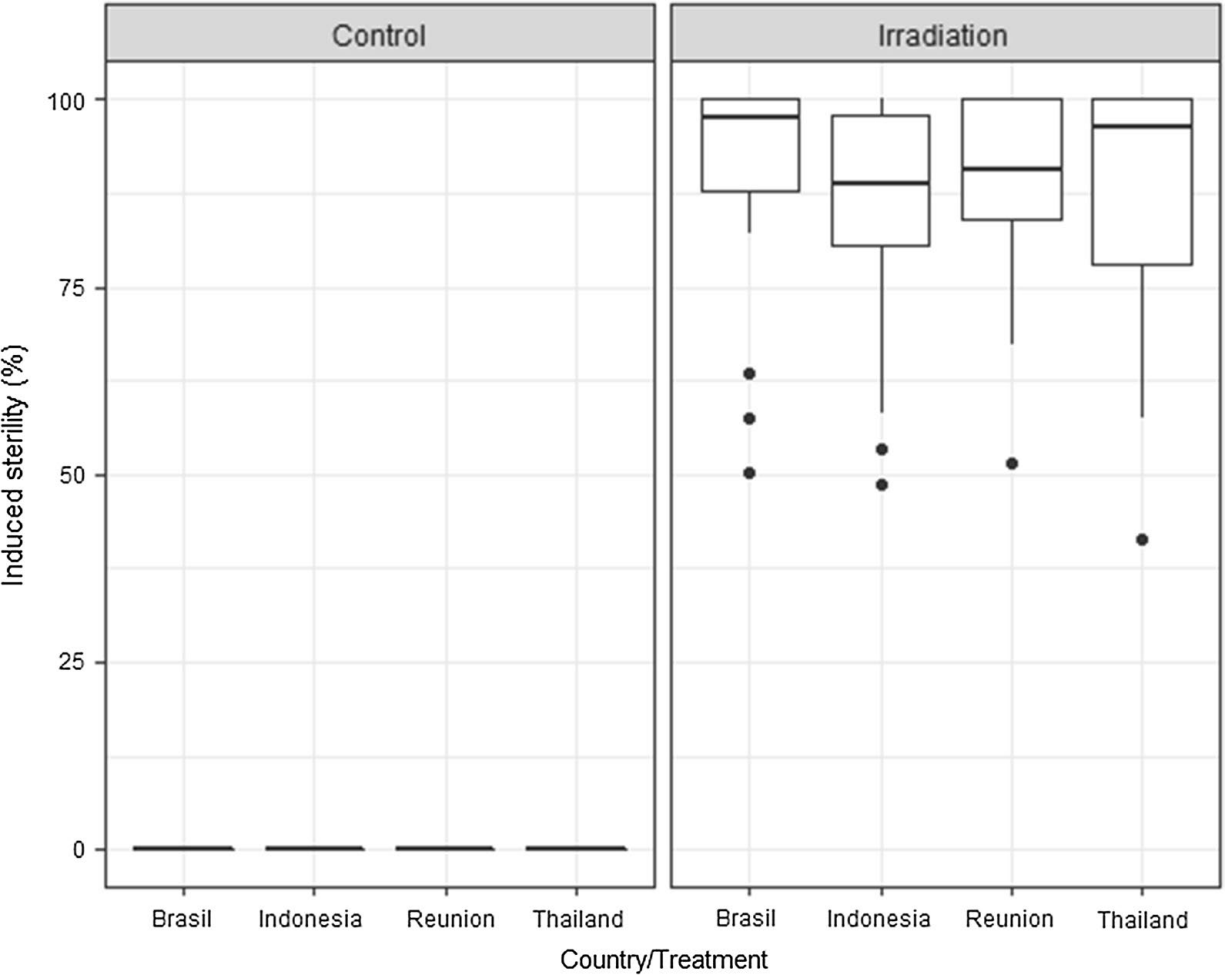
Comparison of radiosensitivity *Ae. aegypti* strains from different geographical origins. No difference was observed following irradiation at 40 Gy. The box plot shows the median and upper and lower quartiles

**Fig. 3:**
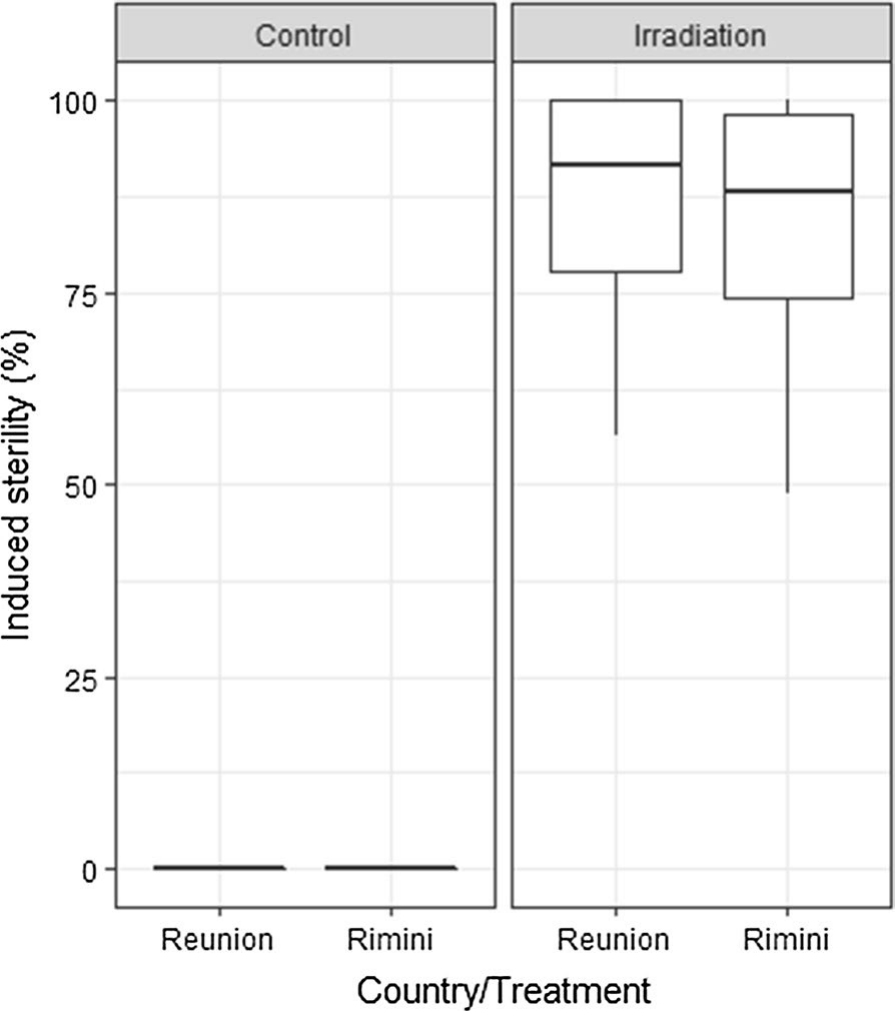
Comparison of radiosensitivity *Ae. albopictus* strains from different geographical origins. No difference was observed following irradiation at 30 Gy. The boxplot shows the median and upper and lower quartiles

There was no significant difference seen in the induced sterility between the 4 strains of *Ae. aegypti* [originating from Brazil, Indonesia, France (La Reunion) and Thailand] [*P* > .05, for all comparisons (*P* = 0.1873, 0.4215, and 0.3919), Fig. 2], nor between the two strains of *Ae. albopictus* (originating from Italy and France (La Reunion); *P* = 0.14, Fig. 3), following irradiation under controlled conditions.

### Effects of age on radiosensitivity and longevity in *Ae. aegypti*

As expected, there was a strong negative correlation between pupal age and radiosensitivity (in terms of resulting induced sterility) (Table 1) with the youngest pupae the most sensitive to irradiation and resistance increasing as the pupal age increases (using median age per group: *R*^2^ = − 0.9513; youngest age per group: *R*^2^ = − 0.9593; oldest age per group: *R*^2^ = − 0.8983).

**Table 1:** Induced sterility in five varying age groups of *Ae. aegypti* pupae following irradiation at 40 Gy

	Control	Pupal age				
		45–50 h	42–45 h	26–42 h	20–24 h	10–24 h
No. of reps	3	2	2	2	2	2
Gy	0	40	40	40	40	40
*n*	1942	507	799	1203	793	1262
avg (HR)	0.80	0.05	0.04	0.03	0.02	0.01
SE	0.01	0.00	0.00	0.00	0.00	0.00
IS	0.00^a^	93.45^b^	95.37^c^	96.44^cd^	96.91^d^	98.93^e^

*Abbreviations*: *n*, total no. of eggs; avg HR, average hatch rate; reps, repetitions; SE, standard error; IS, induced sterility

*Note*: Values followed by different superscript letters are significantly different (*P* < 0.05)

The radiosensitivity was significantly impacted by pupal age (*F*_(5, 7)_ = 34218, *P* < 0.0001). When different age group were compared, old pupae (45–50 h) were more resistant to irradiation compared to all other pupal ages tested in this experiment [*P* < 0.05 for all comparisons (*P* < 0.0001, *P* = 0.0082, 0.00057 and 0.00022, *P* < 0.0001]). Pupae older than 26 h were also more resistant than that of age below 24 h (*P* = 0.0018 and 0.0061).

Results relating to the longevity of adults following irradiation at a fixed dose at pupal stages with varying age are summarized in Table 2. The mean longevity was significantly reduced only for the pupae irradiated at the youngest age (10–24 h; *P* < 0.0001). The number of days for the population to reduce to 50% was only significantly reduced in the youngest age group (*P* < 0.0001), who were generally the poorest survivors amongst the treatment groups.

**Table 2:** Mean longevity and mean number of days required for the population to be reduced to 50 or 0% of the starting population size (*P* threshold value is 0.0033 when comparing all groups)

Pupal age	Days to population reduction			Mean longevity (days)
	50%	90%	100%	(mean ± SE)
Control	23.0	32.6	38.0	19.57 ± 3.0^a^
45–50 h	27.3	36.5	38.0	22.26 ± 3.3^a^
42–45 h	25.5	34.5	38.0	25.40 ± 1.8^a^
26–42 h	23.3	37.2	38.0	22.56 ± 2.3^a^
20–24 h	19.0	34.8	38.0	21.29 ± 2.9^a^
10–24 h	11.0	24.4	28.0	12.81 ± 1.9^b^*

*Note*: Values followed by different superscript letters are significantly different from each other [log-rank (Mantel-Cox) test *P < 0.0033]

### Effects of pupal size on radiosensitivity in *Ae. aegypti*

**Fig. 4:**
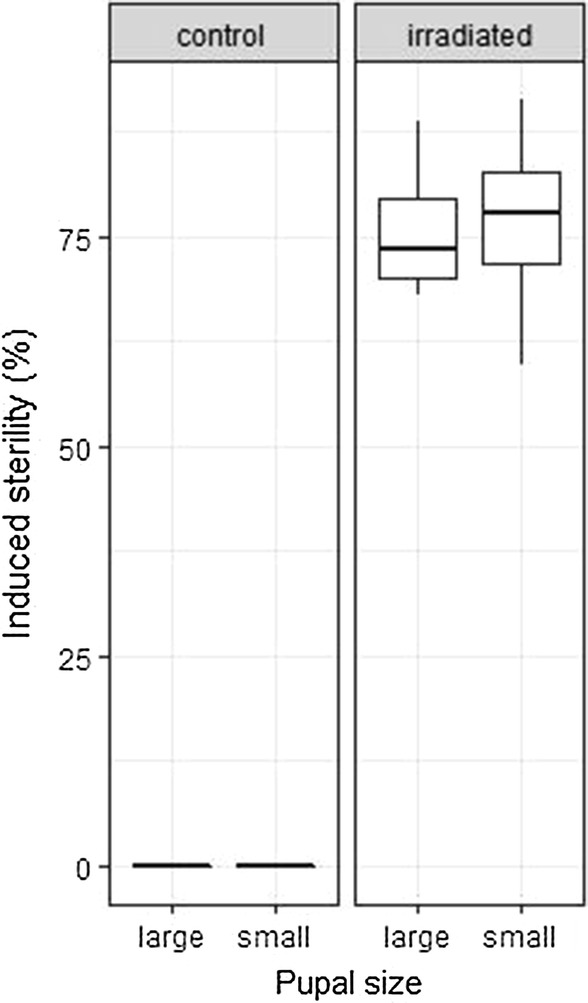
Effects of pupal size on dose-response in *Ae. aegypti* male pupae. No difference was observed following irradiation at 40 Gy. The boxplot shows the median and upper and lower quartiles

Following irradiation at 30 Gy, the variation amongst samples was quite high. However, the mean induced sterility in small and large pupae was 76.5 ± 6.5% and 75.9 ± 4.6% (mean ± SE) respectively (Fig. 4). No difference in radiosensitivity was observed between the samples of small and large pupae (*P* = 0.94).

### Effects of sample preparation and ambient conditions during exposure

**Fig. 5:**
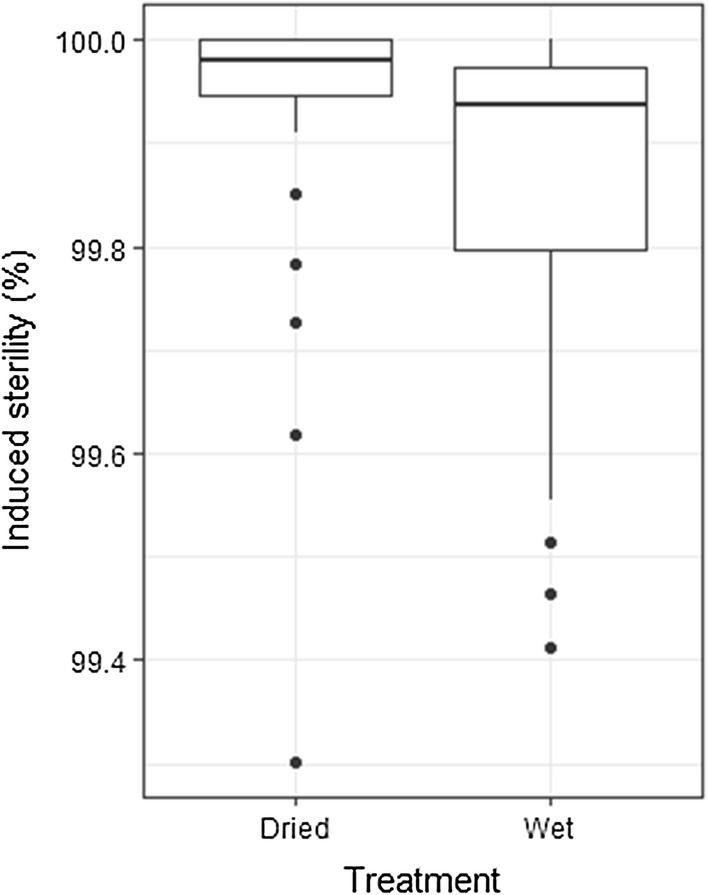
Effects of sample preparation and ambient conditions during exposure. A significant difference was detected between the dry and wet treatments (*P* < 0.005). The box plot shows the median and upper and lower quartiles. Dots indicate outlying data points

Significant differences were seen in dose-response (and resulting induced sterility) following irradiation of the *Ae. albopictus* pupae in controlled conditions, but with differing sample preparation methods (*t*_(104)_ = − 2.87, *P* = 0.0049) (Fig. 5). In addition to this, more variation was observed amongst individual males irradiated in water (wet treatment), although outliers were present in both treatment groups (Fig. 4).

### Effects of hypoxia on induced sterility in *Ae. aegypti*, *Ae. albopictus* and *An. arabiensis*

In all three species, irradiation at the pupal stage significantly reduced the hatch rate (*P* < 0.0001). Moreover, hypoxic conditions showed an attenuation of effects and overall levels of sterility induced (Table 3; *P* < 0.0001). A 3.8-fold increase in residual fertility was seen in hypoxic *versus* normoxic conditions in *Ae. aegypti* after a dose of 70 Gy, with similar trends found for *Ae. albopictus* (at 35 Gy) and *An. arabiensis* (at 95 Gy) with a 2.3 and 3.0-fold increase, respectively.

**Table 3:** Induced sterility in adults following irradiation at pupal stage in either normoxic or hypoxic conditions, compared to unirradiated controls

Strain	Treatment	No. of true reps (tech. reps)	Dose (Gy)	HR ± SD (%)	IS (%)	SE (IS)	SD (IS)
*Ae. aegypti*	Control	4 (3)	0	85.41 ± 0.09	0.00^a^	0.0253	na
	Normoxia	4 (3)	70	1.49 ± 0.01	98.25^b^	0.0021	0.04
	Hypoxia	4 (3)	70	5.67 ± 0.01	93.35^c^	0.0043	0.04
*Ae. albopictus*	Control	4 (3)	0	66.59 ± 5.56	0.00^a^	2.7778	na
	Normoxia	4 (3)	35	14.32 ± 9.12	78.49^b^	4.5574	0.04
	Hypoxia	4 (3)	35	32.30 ± 5.09	51.49^c^	2.5438	0.06
*An. arabiensis*	Control	3 (3)	0	70.32 ± 2.38	0.00^a^	0.8131	na
	Normoxia	3 (3)	95	2.29 ± 0.59	96.74^b^	0.3415	0.01
	Hypoxia	3 (3)	95	6.76 ± 1.41	90.39^c^	1.3763	0.01

*Note*: Values followed by different superscript letters are significantly different *P* < 0.0001. *Abbreviations*: HR, hatch rate; IS, induced sterility; reps, repetitions; SD, standard deviation; SE, standard error; na, not applicable

When hatch rates were corrected by the natural fertility rate measured in the control group [42] to calculate the induced sterility (IS), the significant differences were equally pronounced between the two treatments (Table 3).

There was no correlation of the total number of eggs produced per cage and the treatment for any of the three species, indicating that it is unlikely that different atmospheric treatments on males have any effect on overall fecundity in their female mates. Control hatch rates averaged 85%, 67% and 70% for *Ae. aegypti*, *Ae. albopictus* and *An. arabiensis*, respectively. The results are summarized in Table 3.

## Discussion

For the experiments singling out factors that could affect the dose-response in mosquito pupae, it was important to ensure that other factors that could influence the results were minimized, if not completely eliminated. The primary factor that requires standardization is dose. Therefore, before the series of experiments was initiated, the irradiator (and its irradiation chamber) as well as the dosimetry were characterized and calibrated, and samples were always placed in the same position within the irradiation chamber. Container shape and material also should be consistent for experiments and routine irradiation. For Gamma-ray irradiators, the shape of the canister can improve dose uniformity within the canister and thus also the sample by avoiding areas of higher or lower dose rate. For isotopic irradiators, the material of which the canister is made does not affect dose uniformity but will affect dose-rate (depending on the material and thickness of the canister walls) [2]. For X-ray irradiators, however, the canister material is important for dose uniformity and dose-rate, as different materials can change the photon spectrum by attenuating low energy photons, which then do not reach the sample. Mass attenuation coefficients for elements and various selected materials can be found on the NIST website (https://www.nist.gov/pml/x-ray-mass-attenuation-coefficients). For the irradiation of mosquito pupae in the Gammacell (Co-60 source), a PMMA (polymethylmethacrylate) tube served as the canister wall, and also as the appropriate build-up material during exposures as its interaction with ionizing radiation is near equivalent to water (as are pupae and biological tissue in general). A thickness of the PMMA of 4 mm is sufficient to achieve an electron equilibrium in the chamber and sample, and for this reason it is recommended for routine irradiation work.

In general, *Anopheles* and *Culex* spp. seem to be more radioresistant than *Aedes* spp. In existing literature, doses (gamma-ray) required for > 99% sterility in males of *Anopheles* spp (including *An. albimanus*, *An. arabiensis*, *An. gambiae*, *An. pharaoensis* and *An. stephensi*) are found to range from ~ 80 to 120 Gy [3, 7, 10, 11, 13]. *Culex* spp. males (*Cx. pipiens molestus*, *Cx. quinquefasciatus*, *Cx. tarslis*) have been reported to require doses around 75–150 Gy for full sterility [12, 15, 32], whereas doses reported for *Aedes* spp. (*Ae. aegypti* and *Ae. albopictus*) are generally lower, ranging from 40 to 90 Gy [4, 41, 45]. In these various studies, pupal ages during exposures are not always the same, and other external factors may also widen the dose range reported, such as rearing and handling methods, or nutritional status of the insects. In our studies, the doses required for 99% induced sterility or more are within the ranges reported above, albeit closer to the upper limits of these ranges. This may be due to the fact that we generally used oldest age ranges for the pupae, and the irradiator and its source (activity), rearing methods, larval diets, etc, are of course unique. Although there were differences in dose-response for each of the three species evaluated here, the geographical origin of the strains of both *Aedes* species did not significantly affect their radiosensitivity. Induced sterility in *Ae. albopictus* collected from different localities in northern Italy was also directly compared by Balestrino et al. [4] but these were also found to be similar.

It has been demonstrated in mosquitoes and other insects that radioresistance increases with age [4, 43, 44]. It is therefore important to control for sample age when characterizing a mosquito’s dose-response and to know the variation expected within an irradiated sample when the age amongst individuals varies. When choosing an appropriate dose to attain a target sterility level, the dose should be selected according to the radiosensitivity of the oldest (and thus most resistant) pupae to ensure that the rest of the pupae will achieve at least this level of sterility. Little effect of pupal age was found for three age groups of *Ae. albopictus* pupae in a study by Balestrino et al [4]. Conversely, it has been shown here in *Ae. aegypti*, that the correlation of pupal age and radiosensitivity is quite linear, and differences in age can alter the reported dose-response result in different assessments, despite the implementation of same protocols, irradiation device and mosquito strain. It is therefore necessary to accommodate the age-related radioresistance with higher irradiation doses to achieve the target sterility. It has been shown in other insects that in general, irradiation at later ages, or later life stages increases biological quality of the adult males [3, 30, 45]. However, Helinski & Knols [46] observed that when *An. arabiensis* pupae were irradiated with a fully sterilizing dose, no difference between young and old pupae was observed in terms of adult male competitiveness. Additionally, no noticeable effect on *Ae. aegypti* male competitiveness was observed following irradiation at doses up to 100 Gy when early (19–23 h) and late (42–46 h) pupae were irradiated [37]. In this study, a broader age range was assessed, and a difference in radiosensitivity was detected at 40 Gy (a non-fully-sterilizing dose). Although the resulting male adult competitiveneness was not included in this study, the male longevity data showed that pupae irradiated when older than 24 hours survived equally well compared to the controls. Pupae aged 24 hours or less survived poorly compared to the other groups. However, it is important to note that the dose used was a diagnostic dose of 40 Gy and may not induce the same trend as would be the case for higher doses. This experiment still showed that pupal age during exposures can impact the resulting sterility as well as longevity, and therefore consistency in sample age is essential for consistent sterility results.

Although in general larger particles absorb more radiation (kJ/kg), and so should be more radiosensitive, in mosquito pupae (*Ae. aegypti*) there were no observable effects of pupal size on dose-response. Most likely, the differences in mosquito pupae size are generally too small to create an effect. However, small differences in preparation and handling methods of the pupae samples can affects dose-response. Pupae placed in water are less sensitive to irradiation than pupae that are placed in a dry environment, i.e. where all water has been removed. Furthermore, there was more variation in induced sterility within the wet sample group compared to the dry treatment samples, where induced sterility was more uniform. One reason may be that pupae swimming in water are partially submerged, and oxygen levels in the water are lower than in air (~ 8% at insectary water temperatures, compared to 21% in air; https://www.engineeringtoolbox.com/air-solubility-water-d_639.html), partially protecting the pupae from irradiation effects [53]. In which case, irradiation on the surface of water cannot ensure a standard exposure of all pupae in a given sample. To further test the hypothesis that pupae submerged in water are protected due to decreased in oxygen saturation, as was observed in the mass-irradiation for *Ae. albopictus* pupae (in water) in the Wolbaki mosquito mass-rearing facility in Guangzhou, China (Zhang et al., unpublished data), it was decided to further investigate this factor in a follow-up experiment where the oxygen levels were the focus.

In this next experiment, submerging pupae in water in a closed container aimed to create a low oxygen environment [hypoxic, in this case referring to a starting DO (dissolved oxygen) level of ~ 8%]. We suspect that this initial DO further decreases as pupae continue to absorb O_2_, presumably by cuticular respiration [54, 55] (Zhang et al., personal communication). We hypothesize that it is the reduced oxygen saturation in the sample environment during irradiation that reduces the irradiation effects, resulting in less sterility induced in the pupae. Hypoxia is known to have a radioprotective effect. This has also been shown in other insect species, although most reports described the irradiation exposure of insect samples in nitrogen. The radioprotective effect of hypoxia in insects requires higher doses to achieve lethality and full sterilization [47]. A low oxygen environment induces damages at the cell level, which leads to an increase in the antioxidant defence mechanisms and enhances the antioxidant capacity during hypoxia to prepare the cell for reoxygenation and minimize oxidative stress [48]. This cellular response that prepares cells for reoxygenation may be one of the crucial factors contributing to the increase in radioresistance. This effect was demonstrated by a study conducted with moths, which compared different developmental stages in hypoxia and normoxia. They showed that for all stages, the individuals that were subjected to hypoxia showed better survival compared to those kept in normoxic conditions [49, 50]. To further verify our hypotheses, that pupae absorb oxygen when submerged in water, thus creating an oxygen-poor (or hypoxic) environment, and that this reduced level of DO has a radioprotective effect in irradiation, we have initiated further studies in the pupae of three species of mosquitoes.

In the present study, we found that the fecundity of the females mated to males irradiated in either atmospheric condition was not affected, and no trend was apparent throughout the repetitions or the strains. This was expected as all females were likely mated and egg production generally relies on the biology of the female. However, we found significant reductions in induced sterility in males irradiated in water in all three species tested. This was most pronounced in *Ae. albopictus* following exposure at 35 Gy with more than a 30% reduction, followed by *An. arabiensis* and *Ae. aegypti* with a 5 and 6.6% reduction in induced sterility, respectively. The large difference seen in *Ae. albopictus* may be due to the overall low hatch rate in this particular cohort. The strain is usually reported to have a hatch rate of over 75% [4, 51]. On the contrary to expectations and past experience, residual fertility was high following exposure at 35 Gy. The reduced hatch in controls *versus* the high fertility in treatment groups may be due to poor storage condition of the eggs, or due to *ad hoc* diapausing behaviour of the females and needs to be further investigated. However, the differential response to irradiation in the two atmospheric conditions was clearly demonstrated and has been reproducible in further studies (data not shown). This effect has also been observed in the operational setting at the Wolbaki mosquito facility. Pupae of *Ae. albopictus* are mass-irradiated (up to 250,000 pupae) in a container filled with water. It was noticed that a higher dose was required to achieve the target sterility levels than was needed for routine, small-scale irradiation in air (D. Zhang, unpublished data). This shows the necessity to reassess dose-rate and dose by dosimetry, and dose-response behaviour in mosquito pupae following any changes in protocols.

Further studies are needed to assess the impact of the radioprotectant properties of hypoxia during irradiation on resulting male biological fitness and to evaluate the advantages and disadvantages of irradiating in water (pupae), nitrogen (adults) or in air with respect to operational practicality and adult sterile male mating propensity and competitiveness in the field.

## Conclusions

Differences in pupal age and changing handling methods and atmospheric conditions have major impacts on the resulting sterility reported for mosquitoes. This demonstrates the need for further clarification in larger-scale trials for the development of standardized methods for treatment prior to- and during radiation exposure with the aim to reliably sterilize male mosquitoes, and to produce competitive males for field releases to control populations of these important disease vectors. Next, we aim to re-evaluate full dose-response curves for the three species of mosquitoes following irradiation in hypoxic *versus* normoxic conditions.

## Data Availability

The datasets used and/or analyzed during the present study including all dosimetry reports are available from the corresponding author upon reasonable request.

## Abbreviations

DO: dissolved oxygen
FAO: Food and Agriculture Organization
HR: hatch rate
IAEA: International Atomic Energy Agency
IPCL: Insect Pest Control Laboratory
IS: induced sterility
NIST: National Institute of Standards and Technology
PMMA: polymethylmethacrylate
RF: residual fertility
SIT: sterile insect technique
